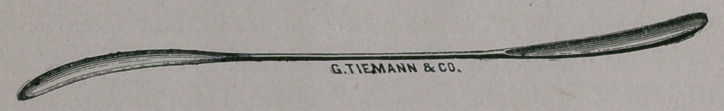# Hints Relative to Intra-Uterine Medication*Read before the American Gynecological Society, 1879.

**Published:** 1880-07

**Authors:** James P. White

**Affiliations:** Professor of Obstetrics and Gynecology, University of Buffalo


THE BUFFALO

MEDICAL AND SURGICAL JOURNAL.

VOL. XIX.—JULY, 1880.—No. 12.

### ORIGINAL COMMUNICATIONS.

### HINTS RELATIVE TO INTRA-UTERINE MEDICATION.*

BY JAMES P. WHITE, M. D.,

Professor of Obstetrics and Gynecology, University of Buffalo.

IT is not the purpose of this paper to consider the pathology or therapeutics of intra-uterine diseases, but to point out simply some of the means which have, in the writer's experience, been found valuable and important in the proper application of remedies to the surfaces within the neck and body of the uterus in diseased conditions already recognized. It is believed that a somewhat detailed description of these measures will be found acceptable to all practitioners in this important department of medicine.

In making applications of fluid substances to the uterine cavity, the most simple method would appear to be by injection, and this method is still advised in the treatises and periodicals of the day. On page 159 of the *Obstetrical Journal of Great Britain and Ireland* for June, 1879, the injection of carbolic acid and water, pure nitric acid, and other liquids is advised in various diseases of the cavity of the uterus. While the most potent

*Read before the American Gynecological Society, 1879.

caustics, as fuming nitric acid, might be applied in full strength to the mucous membrane of the neck or body of the uterus, modifying the condition of the diseased surfaces without exciting grave symptoms, no liquid, however bland, can be injected into the uterine cavity without the liability of exciting terrific uterine colic, if nothing more serious. The experienced practitioner seldom or never injects medicinal agents into the cavity of the uterus. It becomes, therefore, important often to enlarge the canal of the neck, even when of normal size, in order that medicaments may be applied to the lining membrane of the body when in a morbid condition, and in stenosis or contraction of this canal the dilatation becomes absolutely necessary.

The means most commonly resorted to for the purpose of dilatation is the employment of tents, made so as to be introduced when dry and compressed, the absorption of moisture producing expansion and dilating the canal. Believing the tents made of sponge preferable, all things considered, to those made of any of the various substitutes, attention will be directed to that variety only.

The tent, as ordinarily made, is exceedingly imperfect, and is frequently made from coarse sponge which has scarcely tenacity enough to hold together. Good soft sponge, of uniform consistence, should always be selected for this purpose. They should be nearly cylindrical in shape, so as to dilate the canal of the neck uniformly, slightly conical at the point to facilitate introduction, and about one and three-fourths inches in length. Each tent should have a longitudinal perforation at its base to receive the instrument, to be described hereafter, for introducing it. More important than all, they should contain in the centre, running quite to the small and internal end, and securely fastened thereto, a small twine or wire, preferably the latter, the end of which should pass out of the base of the tent to sufficient length to be easily grasped in removing it. Securing the cord or wire quite at the extremity is of the highest importance. It has frequently occurred to the writer to have the tent part in the middle, when making an attempt to remove it, by the twine fastened in the usual way only at or near its base. Few things are more embarrassing to the operator than to find himself called upon to remove the upper half of a tent thus retained at the os internum. Forceps, introduced however carefully, can scarcely be opened and fixed upon the fragment, or, if it be seized, owing to its friable nature, the operator is obliged to bring away a small portion at a time. Failing to seize the retained portion, it is pushed in front of the forceps into the uterine cavity, necessitating complete dilatation of the os and neck before it can be secured and removed.

A single case will be sufficient to illustrate an annoyance which can easily be avoided by proper arrangement of the string or wire, but which was the result of using a sponge tent as at present constructed.

Miss B, aged 18, with severe dysmenorrhea, accompanied with profuse catarrhal discharge from the os, had a moderate-sized sponge tent introduced in the evening, which was found fully expanded the following morning. A cylindrical speculum was introduced and moderate traction made on the string attached to the base of the tent, but only about three-fifths of the tent was drawn out. In an effort to extricate the retained fragment, it was pushed forward into the cavity, and could not be removed without further expansion of the canal. The forceps could be introduced into the os and passed up to the retained sponge, but could not be opened so as to grasp it. Menstruation being near at hand, it was deemed wise to omit further efforts towards its extraction until after that period had passed. The flow came on a day or two subsequently, was free, and without any of the terrible suffering to which the patient had been accustomed. Her general health rapidly improved, and the patient persistently refused to permit any further efforts to be made for the removal of the retained sponge, and after a full explanation of the annoyance and danger which it would be certain to occasion, returned to her home in Canada, some hundred miles distant.

In about six months she returned for its removal, assuring me that notwithstanding all her efforts at cleanliness with the free use of deodorizing vaginal injections and washes, the discharge had become so offensive and profuse that she was obliged to seclude herself, and her own family would not tolerate her presence.

A large tent was introduced in the afternoon, and the following morning the canal was so fully dilated that I was enabled to seize the specimen, which I here show you, and remove it without pain or difficulty.

The piece of sponge here exhibited is useful in illustrating the little change that had taken place during its retention of more than six months in the uterine cavity, and how futile would be any delay in expectation of its disintegretion or expulsion by uterine contraction.

Other similar cases could be cited, but this is quite sufficient to show how important it is that the wire or twine for the extraction of sponge tents pass entirely through them, and be secured at their apex so as to command the entire mass of expanded sponge.

The tent may be bent to accommodate its form to the flexions which may be present. It may be covered with gold-beater's skin, with tin foil, or with some gelatinous material, to facilitate introduction and to prevent irritation of the mucous membrane, but not with tallow or rancid cerate. By constructing the tent of sponge, as here described, in accordance with the specimens exhibited, it becomes a safe means of overcoming stenosis, and the operator will not be liable to meet with the accident just described.

It has already been stated that the tent should have a perforation at its base, into which the point of the tent holder could be inserted. It will readily be seen that the operator has much better control of the tent by this holder than when held by forceps, as often recommended. The movable coil of wire around the stylet enables the operator to dislodge the tent, when in position, without in the least disturbing its relation to the parts. The instrument should be bent to conform somewhat to the pelvic curve. The whole procedure may be made through a cylindrical or Sims' speculum, or upon the finger without either. Sims' hook, with a long handle, is often very useful in holding the uterus forward and straightening the canal during introduction. The hook, if properly used, seems also to help the operator to pull on, as it were, the glove.

It will often be found that time will be saved in the process by incising the lining membrane of the neck before any attempt is made to dilate. It is not necessary to make deep incisions, nor should they be as superficial as recommended by the lamented Peaslee in an article on stenosis, written shortly previous to his death. These incisions are best made with the long, slender blades here shown, and their depth is to be governed by the skill and judgment of the operator, and not by a mechanical hysterotome.

By resorting to these incisions, the canal is rendered much more dilatable, the endo-metritis of the neck lessened, and if prudently made, there is no danger of hemorrhage or pelvic abscess.

Whether the process of dilatation be preceded by tents or by the knife, or both, sometimes without either, the dilator here exhibited may be frequently used to dilate the canal or keep it patulous.

No matter by what process the dilatation is primarily accomplished, the canal is almost certain to contract, and even to become narrower than prior to treatment, unless regularly dilated for a considerable period subsequent to the first operation. The instrument here shown possesses many advantages over any other with which I am familiar. It is as easily introduced as Simpson's sound. The dilating force can be applied as gently as desired; *it is elastic and continuous, and may ordinarily be continued and increased for any length of time without pain.* This instrument, which I have now used for more than thirty years, is very simple in construction and inexpensive. The amount of pressure is regulated with a screw, and is entirely under the control of the operator. Having dilated the canal, and the passage of instruments of moderate size now being practicable, we shall find the long probe of hard rubber or whale-bone, first used, I believe, by Professor Miller, of Louisville, Ky., and by him called an applicator, very useful. The point is easily coated with cotton or muslin, which can be saturated with any desirable medicament, and applied to the uterine membrane. The cotton or muslin is then easily removed and fresh material substituted. Thus armed, this probe is very convenient for removing from the membrane the catarrhal coating which absolutely prevents the application of substances to the surface until removed. The rag or cotton may be saturated with vinegar, which will coagulate the albuminous secretion and facilitate its removal. It may be here remarked, also, that acetic acid or common vinegar should always be at hand for removing sanguinolent or other matters which interfere with inspection or treatment of the os or canal. Vinegar is a good astringent, coagulates, as already remarked, the albumen, removes muco-purulent discharges, and does not discolor the surface to which it is applied. Hence, in an examination with cancer or epithelioma in a hemorrhagic condition, dossils of cotton saturated with vinegar, applied to the surfaces on a probe or in forceps, will be found exceedingly convenient. The applicator or probe above described is not only useful in applying various remedies to the uterine mucous membrane by means of cotton or muslin saturated with these and wrapped about its point, but it may be coated for a short distance with nitrate of silver, deposited upon its surface by crystallization.

For many years I have been accustomed to use nitrate of silver, either in substance in the ordinary crayon, or the still milder crayon used by oculists, or the points of Squibb, secured in rubber tubing. Notwithstanding that I have caustic-holders of gold, platinum, hard rubber, and various other materials, for many years, I have used exclusively the rubber tubing of various sizes. It holds the crayon firmly, is so flexible that, bending at the point of junction between the forceps or staff and the pencil, it adapts itself to the flexions of the canal without fracture of the crayon, as would be apt to occur in a rigid holder. This form of holder affords no opportunity for the instrument maker to display his taste or fill his exchequer, being almost without cost and readily made by any novice. The crayon and rubber holder may be slipped into a larger tube, taking the precaution to insert a stick alongside to prevent bending and fracture, and in this way it may be conveniently carried in the pocket and be always ready for use. This arrangement is unexceptionally safe and convenient, but lacks the attractiveness of more expensive paraphernalia, and will never be introduced to professional notice by the instrument makers.

Glass tubes or rods drawn to a point, similar to the extremity of the uterine probe, may be roughened and made a useful vehicle for carrying caustic fluids into the neck of the uterus, and there applying them. Sufficient nitric acid or saturated solution of chromic acid, or similar caustics, will adhere to this ground glass applicator for an ordinary application to the mucous membrane. Glass is for many reasons superior to all other materials for the handling and transmission of caustics, when it can be used, and it is inexpensive. But the cavity of the uterus cannot be reached with fluid on these glass or rubber probes. The narrow canal of the neck wipes off or removes any medicament on the surface of the probe, and it reaches the body of the uterus after parting with its surface coating in its passage. As has already been stated, injections, properly so-called, are inadmissible. Many instruments, uterine specula, canulæ, etc., have been devised for carrying caustics and other remedies up to the cavity of the uterus, without in their passage unnecessarily coming in contact with the membrane lining the canal of the neck.

After much reflection and many unsatisfactory trials of various methods of overcoming the difficulty, I made a trial of a small glass tube, drawn out at one extremity and curved in the form of the uterine sound. In this tube I could carry any desired number of drops of fluid by simply dipping its extremity to the proper depth in the liquid. By placing my finger or thumb over the opposite end, the fluid was easily retained as long as the air was excluded. The tube thus charged was carried up through the neck, and by careful manipulation its contents deposited upon the membrane lining the cavity. This procedure was somewhat difficult, and required the exercise of great care not to allow the finger to be prematurely removed from the end of the tube, and thus deposit the fluid upon surfaces where it was not desired. Some years since I placed the small rubber air-bulb which was found on the end of a pipet-drop glass over the end of my long tube, and secured it there by means of twine. This arrangement proved eminently satisfactory. By means of the bulb I could easily draw up any desired number of drops, which were retained until made to exude by gently compressing the bulb. The point of this glass tube may, by a little gentle movement, be carried to different parts of the membrane, and the fluid deposited or spread over the surface. This method of applying substances cannot properly be called an injection any more than when applied by a sponge or dossil of cotton. The inner surfaces of the neck are not touched by the fluid, as in carrying up the armed probe ; more than that, you can deposit a definite number of drops with almost perfect accuracy in any desired locality. Again and again have I carried up a definite number of drops, from three or four to thirty, of fuming nitric acid, and deposited them gradually upon the inner surface of the uterine cavity. It is proper here to remark that, while I am not an advocate of the frequent resort to this heroic treatment, when used as here described, I have never known it productive of serious consequences. It is astonishing with what impunity the most powerful caustics may be thus applied to the inner surface of the uterus, while the most bland and anodyne solutions cannot be injected into the cavity without, in a certain proportion of cases, producing the most alarming symptoms. All gynecologists are aware that the potential cautery can be applied to the os and neck with but little pain, and is rarely followed by grave symptoms, although by no means always producing the desired result. The same rule will hold true with the application of caustics in fluid form, providing they are used in the manner indicated. After several years of trial of this simple glass instrument, I am prepared to say that I believe it indispensable in the treatment of intra-uterine disease, and commend it to the favorable consideration of members of this Society. Many friends have been induced to make trial of it during the last few years, and with but one voice lauded its utility. It may be used to carry up any liquid substances deemed desirable for introduction into the uterine cavity.

It need scarcely be again remarked that its contents must invariably be dropped, and not expelled or injected. This is easily regulated by graduating the pressure on the rubber bulb.

It remains to call attention to another method of intra-uterine treatment which is often found of incalculable service in arresting hemorrhage or serous discharges in granular or polypoid developments on the uterine mucous membrane.

The curette of Recamier is, in the writer's opinion, far superior to the "modern improvements" or substitutes. The instrument recommended by Dr. Sims is too small, and is utterly inefficient. Peaslee's may answer in some instances, but I fail to perceive that it is in any respect superior to the one originally recommended by Recamier, and the same may be said of all the modifications introduced by various operators. The instrument can be much more conveniently used if made, as here shown, longer than originally designed. The edges, while they should have sufficient sharpness to remove the bead-like growths studding the membrane, should not be so sharp as to endanger the deeper structures.

The danger of deep wounds is guarded against by curving well the inner or cutting edge of the spoon-like margins.

In these desultory remarks it has been my object to call attention to methods and means which in my hands have proved of value in the treatment of intra-uterine disease. They are not hastily recommended to the Society, but are the result of long and careful observation.

Indeed, this article, calling attention to the instruments and appliances herein described, is written in compliance with a request of some of the oldest and most successful practitioners among the Fellows of this Society, and without claiming that all the measures here recommended are new to the profession.

## Figures and Tables

**Figure f1:**
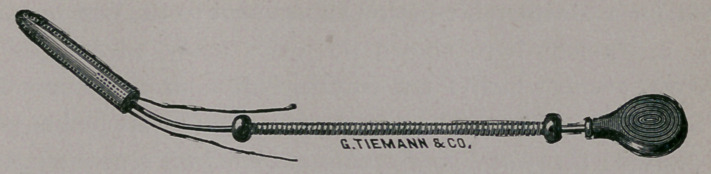


**Figure f2:**
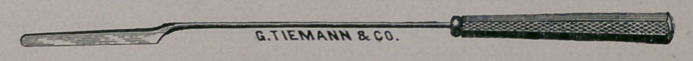


**Figure f3:**
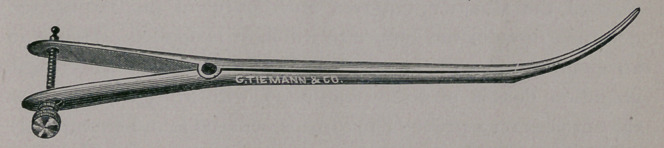


**Figure f4:**
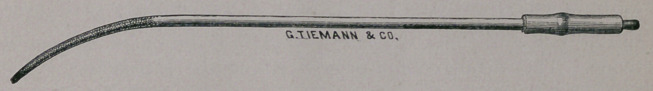


**Figure f5:**
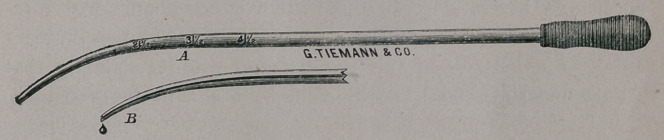


**Figure f6:**